# The Effectiveness of Positive Psychology Interventions for Promoting Well-being of Adults Experiencing Depression Compared to Other Active Psychological Treatments: A Systematic Review and Meta-analysis

**DOI:** 10.1007/s10902-022-00598-z

**Published:** 2022-11-05

**Authors:** Wei Loong Lim, Stephanie Tierney

**Affiliations:** 1 Department for Continuing Education, University of Oxford, Oxford, UK; 2 Nuffield Department of Primary Care Health Sciences, University of Oxford, Oxford, UK

**Keywords:** Positive psychology interventions, Depression, Well-being, Happiness, Systematic review, Meta-analysis

## Abstract

This systematic review assesses if positive psychology interventions (PPI) are more effective than other active psychological interventions for increasing the well-being of depressed adults. A review of randomised trials that compared PPI to other active interventions was conducted. A systematic search was undertaken using PsycInfo, PubMed, EMBASE, Web of Science, Scopus, CINAHL, two trial registries, and a manual search. The outcomes were happiness and depression. Ten studies, totalling 1341 participants, were included in the review. The small effect sizes for depression (Hedge’s g = 0.15) and happiness (Hedge’s g = 0.20) favoured PPI but were not significant, indicating no difference between PPI and other active interventions for the outcomes. Heterogeneity was high mainly due to differences in trial implementation. Risks of bias ranged from moderate to high. The results should be interpreted with caution because of the small number of included studies, high heterogeneity, and presence of bias.

*Protocol Registration Number* PROSPERO CRD42019152513.

**Supplementary Information:**

The online version contains supplementary material available at 10.1007/s10902-022-00598-z.

## Introduction

Positive psychology sets itself apart from other psychological interventions by claiming that rather than merely treating mental health conditions, it builds positive resources that foster well-being (Seligman et al., 2006). A systematic review of randomised trials was planned to assess if positive psychology is more effective than other active psychological interventions for increasing the well-being of adults experiencing depression. Existing systematic reviews that relate to this topic have included studies using non-treatment or waitlist comparators (Bolier et al., 2013; Carr et al., 2020; Chakhssi et al., 2018; Hendriks et al., 2020; Sin & Lyubomirsky, 2009). In contrast, the present review only included trials that compared positive psychology interventions (PPI) to other active interventions.

Globally, more than 264 million people are affected by depression, making it one of the leading causes of disability (World Health Organisation, 2020). Yet an estimated 76% and 85% of people with depression in low- and middle-income countries respectively receive no treatment (World Health Organisation, 2020). The reasons could be non-availability or lack of access to treatment, or social stigma. The COVID-19 pandemic has exacerbated this situation. COVID-19 has affected people’s mental health globally (Waters et al., 2021; Xiong et al., 2020). However, those who are affected might not be able to access timely mental health support due to lockdowns and service closures. It has thus become more pressing to find an effective intervention that can be self-administered or delivered remotely. In this regard, compared to other active interventions, PPI is low-cost and low intensity; hence it can be applied on a wider scale with less resources. It is therefore worthwhile comparing PPI’s effectiveness to other active interventions to ascertain its viability as an alternative approach to supporting people living with mental health conditions.

## Background

### Positive Psychology and Increasing Well-Being

The positive psychology movement emerged in the wake of psychology’s overwhelming emphasis on pathology. Seligman and colleagues (Csikszentmihalyi & Seligman, 2000; Seligman et al., 2006) observed that since the end of the Second World War, research in psychology had been dominated by the study of mental illness. They argued (Csikszentmihalyi & Seligman, 2000; Seligman, 1999; Seligman et al., 2006) that while it was important to study pathology, this almost exclusive focus on diseases and their treatments benefitted only a minority of people suffering from mental health conditions. Seligman and his colleagues saw this as veering away from psychology’s original mission of bettering the lives of all people, hence their launch of the positive psychology movement.

The positive psychology movement quickly gave rise to the development of PPI—an umbrella term for activities that promote positive thoughts, emotions and behaviours with the long-term goal of contributing to psychological growth and well-being (Sin & Lyubomirsky, 2009; Sin et al., 2011; Schrank et al., 2014, 2016; Sutipan et al., 2017; Hendriks et al., 2018). The form PPI takes ranges from one single activity, such as gratitude journaling or performing an act of kindness (Kerr et al., 2015; Otake et al., 2006), to a multi-component intervention, such as the Positive Psychotherapy programme (Seligman et al., 2006; Rashid, 2015), which consists of a series of activities organised into 14 sessions.

Over the last three decades, evidence supporting PPI’s effectiveness has been accruing. PPI has been shown to both reduce depression and promote well-being and optimal functioning for the general population, mental health groups, and patients suffering from chronic or terminal illnesses. Sin and Lyubomirsky (2009) published the first systematic review on PPI’s effects on depression and well-being. They included 51 interventions in their meta-analysis, which yielded significant medium effect sizes of r = 0.29 and 0.31 in favour of PPI for improved well-being and reduced depression. Sin and Lyubomirsky (2009) interpreted the results as evidence of PPI’s effectiveness. However, they noted significant heterogeneity among their studies, which they addressed by analysing moderator effects. The moderator analyses showed PPI to work better for participants with depression compared to non-depressed participants, although this could be the result of a flooring effect. PPI was more effective for self-referred participants and older participants. It also worked better when delivered as individual therapy compared to group and self-administered formats.

Sin and Lyubomirsky’s (2009) lack of quality appraisal and their inclusion of quasi-experimental designs might have resulted in their effect sizes being overestimated due to lower study quality. Bolier et al. (2013) conducted a similar review but selected only randomised controlled trials (RCT). They also critically appraised their included studies to assess risk of bias. They meta-analysed 39 studies and found significant small to medium effects of d = 0.34, 0.20, and 0.23 for subjective well-being, psychological well-being, and depression respectively. The effect sizes were reduced at follow-up assessments, although still significant. Bolier and colleagues cautioned that the quality of most studies were low to medium (20 low, 18 medium, 1 high), so even with the modest effect sizes, they could still have been inflated. Their review found moderator effects, two of which echoed the results of Sin and Lyubomirsky (2009). These were larger effects found for individual-based interventions and among participants with specific psychosocial problems. In addition, Bolier et al. (2013) found larger effects for longer duration interventions and for participants who were recruited through hospitals or healthcare professionals.

Hendriks et al.’s (2020) systematic review, which focused on the effects of multi-component PPI, also selected RCTs only. The meta-analyses of 50 studies yielded small to moderate effect sizes for subjective well-being (g = 0.34), psychological well-being (g = 0.39) and depression (g = 0.32). However, compared to Boiler et al., there were more studies in Hendricks et al.’s review that were assessed to be of moderate and high quality (13 high, 21 moderate, 16 low), and the effects were reduced when the low quality studies were removed from the analyses.

In another review by Hendriks et al. (2018) on the efficacy of PPI in non-Western populations to evaluate the cross-cultural validity of PPI, the reviewers meta-analysed 28 RCTs mostly conducted in Middle Eastern and East and South Asian countries. Effect sizes at post-treatment were moderate to large for all outcomes vis-a-vis the mostly smaller effect sizes in Western studies. However, the authors cautioned that most of the reviewed studies were of low quality (23 low, 2 medium, 3 high quality), and heterogeneity was significant in all the comparisons. Hence, the effects were most likely overestimated.

Finally, in their systematic review, Carr et al (2020) attempted to overcome the shortcomings of previous reviews by setting more inclusive eligibility criteria. They included clinical and non-clinical populations, as well as various PPI types and format, different mental health conditions, age groups (including children), countries (including non-western countries), and publication types and publication languages. This yielded 347 included studies with more than 72,000 participants. The meta-analyses produced medium effects for well-being (g = 0.39) and depression (g = −0.39). With regard to study quality, as with previous reviews, the included studies were mostly rated as fair (152 studies) or low in quality (164 studies).

By including both general and clinical populations in their reviews, Sin and Lyubomirsky (2009), Bolier et al. (2013), Hendricks et al. (2020; 2018) and Carr et al. (2020) provided a broad-spectrum view of PPI’s effectiveness. Other syntheses have focused on specific groups. For examples, Schrank et al.’s (2014) narrative literature review discussed how PPI supported recovery from mental health conditions. Likewise, Walsh et al. (2017) conducted a systematic review on studies that included only individuals who had been formally diagnosed or had met the assessment criteria for depressive or psychotic disorders. Santos et al.’s (2013) systematic review addressed PPI’s effectiveness on treating depression. Generally, the results of these reviews converged on PPI being effective in reducing negative symptoms and increasing well-being. Meta-analysis was not conducted in these reviews. On the other hand, Chakhssi and colleagues (2018) meta-analysed 30 studies that tested PPI among clinical populations diagnosed with psychiatric or somatic illnesses. The meta-analyses yielded small effects for increasing well-being and reducing depression, as well as a moderate effect for reducing anxiety. As with other reviews, the quality of studies in Chakhssi et al.’s review ranged from low (n = 18) to medium (n = 12). After removing the low-quality studies, the effect sizes were reduced to non-significant for depression and anxiety.

Thus far, the accumulative evidence from the systematic reviews cited above points to PPI being moderately beneficial for enhancing well-being and reducing depression, and PPI being more effective as individual therapy, over a longer period of time, and when a variety of activities are practiced instead of a single activity.

### Positive Psychology vs. Other Active Interventions

Positive psychology’s major doctrine is that the absence of negativity does not directly imply the presence of positivity (Lee Duckworth et al., 2005; Seligman et al., 2006). In other words, not being mentally ill (the absence of depressive symptoms) does not automatically bring forth happiness (the presence of positivity). Merely recovering from a mental health condition is therefore insufficient if well-being is not gained and enhanced as well. On this premise, positive psychologists distinguish PPI from other standard psychological interventions by stating that while other interventions mainly target negative symptoms, PPI promotes positive thoughts, feelings and behaviours, which in turn creates sustainable recovery and long-term well-being (Lee Duckworth et al., 2005; Seligman et al., 2006). Should this claim be true, one would expect PPI and other active interventions to be equally effective in reducing negative symptoms, but PPI to fare better in promoting well-being. However, to date, systematic reviews examining the effectiveness of PPI have included trials that mostly compared PPI to no-treatment or wait-list (for example, Bolier et al., 2013; Chakhssi et al., 2018; Sin & Lyubomirsky, 2009). Systematic reviews that solely compare PPI with other active psychological interventions are limited and fairly recent (Carr et al., 2020; Geerling et al, 2020).

Furthermore, individual trials that directly compared PPI with another active intervention have shown mixed results. Furchtlehner et al.’s (2019) RCT comparing group-based PPI and group-based Cognitive-Behavioural Therapy (CBT) for treating depression found PPI to fare significantly better. Conversely, another trial by Chaves et al. (2017) showed no difference between group-based PPI and group-based CBT on all outcomes. It would be beneficial to synthesise these and similar studies to clarify the matter. Such a comparison has important practical implications. PPI is relatively low-cost, requires less training to administer, and can be self-administered. Therefore, it can be implemented more cost-effectively and on a larger scale, compared to other active techniques. In situations where it may be costly to provide standard treatments or in resource-deprived places where patients are unable to access standard treatments, positive psychology could be a viable alternative (Layous et al., 2011).

As mentioned, there has not been any systematic review comparing only PPI and another active psychological treatment until recently (Carr et al., 2020; Geerling et al., 2020). In their review, Carr et al. (2020) analysed comparators as moderators. They found smaller effect sizes when PPI was compared to other active interventions (well-being g = 0.31; depression g = −0.30) than when PPI was compared to no-treatment controls (well-being g = 0.55; depression g = −0.52). On the other hand, Geerling et al.’s (2020) review did not find significant differences between PPI and active interventions for both outcomes. However, these two reviews are not comparable. Carr et al.’s (2020) review was wide-ranging as previously mentioned, while Geerling et al. (2020) studied only adults who were suffering from severe mental illness such as major depression, schizophrenia and bipolar disorder.

The wide-ranging focus of Carr et al.’s (2020) review limits its ability to inform specific practical applications. Moreover, it only searched databases until Dec 2018. Furthermore, Geerling et al. (2020) focused on a clinical population, meaning findings are not necessarily transferable to community dwelling adults. The review presented below addressed these issues.

## The Present Study

In light of the foregoing discussion, a systematic review was conducted on the effectiveness of PPI compared to other active psychological interventions for improving the well-being of adults with depression. It excluded studies that used no-treatment, waiting list, or non-active interventions as comparators. It was hypothesised that PPI would be more effective than other active comparators for improving the well-being of adults experiencing depression. It was also hypothesised that there would be no difference between PPI and other active treatments in reducing depressive symptoms. Depression was selected for the review because PPI has most often been used to treat it compared to other mental health conditions.

## Methods

The review was conducted according to the preferred reporting items for systematic reviews and meta-analyses (PRISMA) and the Cochrane Handbook for Systematic Reviews of Interventions’ guidelines (Higgins et al., 2019). Its protocol was registered on PROSPERO, an international register of systematic reviews (registration number CRD42019152513).

### Search Strategy

Electronic database searches were carried out on PsycINFO, PubMed, EMBASE, Scopus, Web of Science, and CINAHL, as well as two trial registers—www.clinicaltrialsregister.eu, and www.clinicaltrials.gov, on 10 April 2019. Updated searches were conducted on 15 November 2019 and 1 May 2020. Text word search terms such as “positive psychology”, depress*, well-being, random*, trial, and their variations were used to search the title and/or abstract fields. Names of individual positive psychology activities (e.g. gratitude, optimism) were also included as search terms. The search strategy varied slightly according to each database’s setting and requirements. Besides the databases and trial registers, references of published reviews (Bolier et al., 2013; Chakhssi et al., 2018; Hendriks et al., 2018; Santos et al., 2013; Sin & Lyubomirsky, 2009; Sutipan et al., 2017; Walsh et al., 2017) were searched. There was no restriction on publication dates.

### Selection of Studies

Eligible studies were selected in two phases. The first was title and abstract screening, and the second a full text review. The first author conducted both phases. Included and excluded studies were then checked by another independent reviewer. Apart from a few minor clarifications on the tools used to assess depression, there was no major disagreement over study inclusion or exclusion. Studies were selected to be included in the review according to the following criteria:

#### Study Design

RCTs with at least two arms, one providing PPI and the other providing another active psychological intervention.

#### Participants

Participants had to be adults (18 + years) and ascertained by validated assessment tools to have clinical or non-clinical depression. Participants must not be receiving institutionalised care for their depression. This is because institutionalised patients would most likely be receiving structured psychiatric treatments that may confound the review’s results. Studies that examined other mental health conditions, such as anxiety disorders and dementia, or included participants with multiple mental health conditions, were excluded. However, studies that included different groups of participants were selected if they included participants that fitted the eligibility criteria and if the outcomes for participants with depression were reported separately and could be extracted for review.

#### Outcomes

The outcomes were well-being and depression. Well-being could be measured as subjective well-being, psychological well-being or happiness.

#### Language

Studies had to be published in English.

### Data Management

Two softwares, *Zotero* and *RevMan*, were used for data management. Initially, all search results were exported to *Zotero*, a reference managing software, to enable offline title and abstract screening, as well as full text review. *Zotero* was also used to identify and merge duplicates before screening. The included studies were then added into *RevMan* for data extraction and analyses.

### Data Extraction

The following data were extracted: (1) participant characteristics (age, gender, depression status), (2) intervention and comparator characteristics (sample size for each arm, activity type, frequency, duration, format), (3) outcomes (types of outcome and methods of measurement), and (4) country in which the trial was conducted. The extracted data were stored in *RevMan*. Eight authors from seven studies (Asgharipoor et al., 2012; O’ Leary & Dockray, 2015; Uliaszek et al., 2016; Broc et al., 2017; Celano et al., 2017; Chaves et al., 2017; Furchtlehner et al., 2019) were contacted to either clarify information or request data. Two responded, five did not, while one could not be reached as the email address listed on the paper no longer worked.

### Risk of Bias

Risk of bias for included studies was assessed using the Cochrane Risk of Bias tool (Higgins et al., 2011). The domains of assessment are sequence generation (selection bias), allocation concealment (selection bias), blinding of participants and personnel (performance bias), blinding of assessment (detection bias), incomplete outcome data (attrition bias), selective reporting (reporting bias) and other biases such as bias as a result of deviation from treatment.

While reviews such as Bolier et al. (2013) took a more conservative approach to critical appraisal, in which non-report of a criterion was given a negative rating, the current review rated similar studies as unclear. This was because it was expected that many behavioural science publications might not follow a standard reporting template (e.g., CONSORT), therefore when a critical appraisal criterion, such as allocation concealment, was not reported, one should not assume that it has not been done; thus an “unclear” rating was deemed appropriate.

### Data Analysis

Post-intervention scores were used for meta-analysis of intervention trials. The meta-analysis for each outcome was conducted using the random effects model as the studies were expected to be heterogeneous. The outcomes were expected to be measured as continuous variables, and by different measurements, hence standardised mean differences (Hedge’s g) were computed as the effect size. Following Hendrik et al.’s (2018) convention, effect sizes of 0–0.32 was considered as small, 0.33–0.55 as moderate, and 0.56–1.2 as large. Positive effect sizes would indicate treatment effects favouring PPI while negative effect sizes would indicate treatment effects favouring comparators.

Depression and well-being were expected to be measured with multiple measures. In such situations, when studies used more than one measure to assess the outcomes, the measures to be used for meta-analysis were selected based on conceptual similarity. This was so that the conceptual integrity of the construct could be preserved. In this way, the results could be interpreted more meaningfully.

Heterogeneity was assessed with the Q statistic and I^2^ statistics. A statistically significant Q statistic at *p* = 0.05 indicates heterogeneity among the studies. As for the interpretation of I^2^, Higgins et al’s (2019) convention was adopted, where:

- I^2^ = 0–40%: might not be important;
- I^2^ = 30–60%: may represent moderate heterogeneity;
- I^2^ = 50–90%: may represent substantial heterogeneity;
- I^2^ = 75–100%: considerable heterogeneity.

Sensitivity analyses were performed to examine if the main results were affected by studies with small sample sizes and studies that did not fully meet the selection criteria but were included in the review. This was done by repeating the meta-analyses with such studies excluded. In addition, as recommended by Higgins et al. (2019), the fixed effects and random effects models were compared to test for small studies effect.

Publication bias was assessed by a funnel plot diagram. An asymmetry on the funnel plot suggests the presence of publication bias. Asymmetry was also assessed using Egger’s test. The funnel plot and Egger’s test are the two tests of publication bias recommended by Higgins et al. (2019) and Sterne et al. (2011) to be sufficient for assessing publication bias. More importantly, instead of relying on post-hoc statistical tests, Sterne et al. (2011) stressed the importance of conducting a systematic and comprehensive search to minimise publication bias. In this review, publication bias was addressed with a more extensive search than previous reviews. It searched six databases compared to fewer databases searched by other reviews. It also searched two trial registers to check for unpublished trials. Names of individual PPI activity were used as search terms to further expand the search. However, publication bias could still exist because we did not search for grey literature.

## Results

### Study Selection

**Fig. 1:**
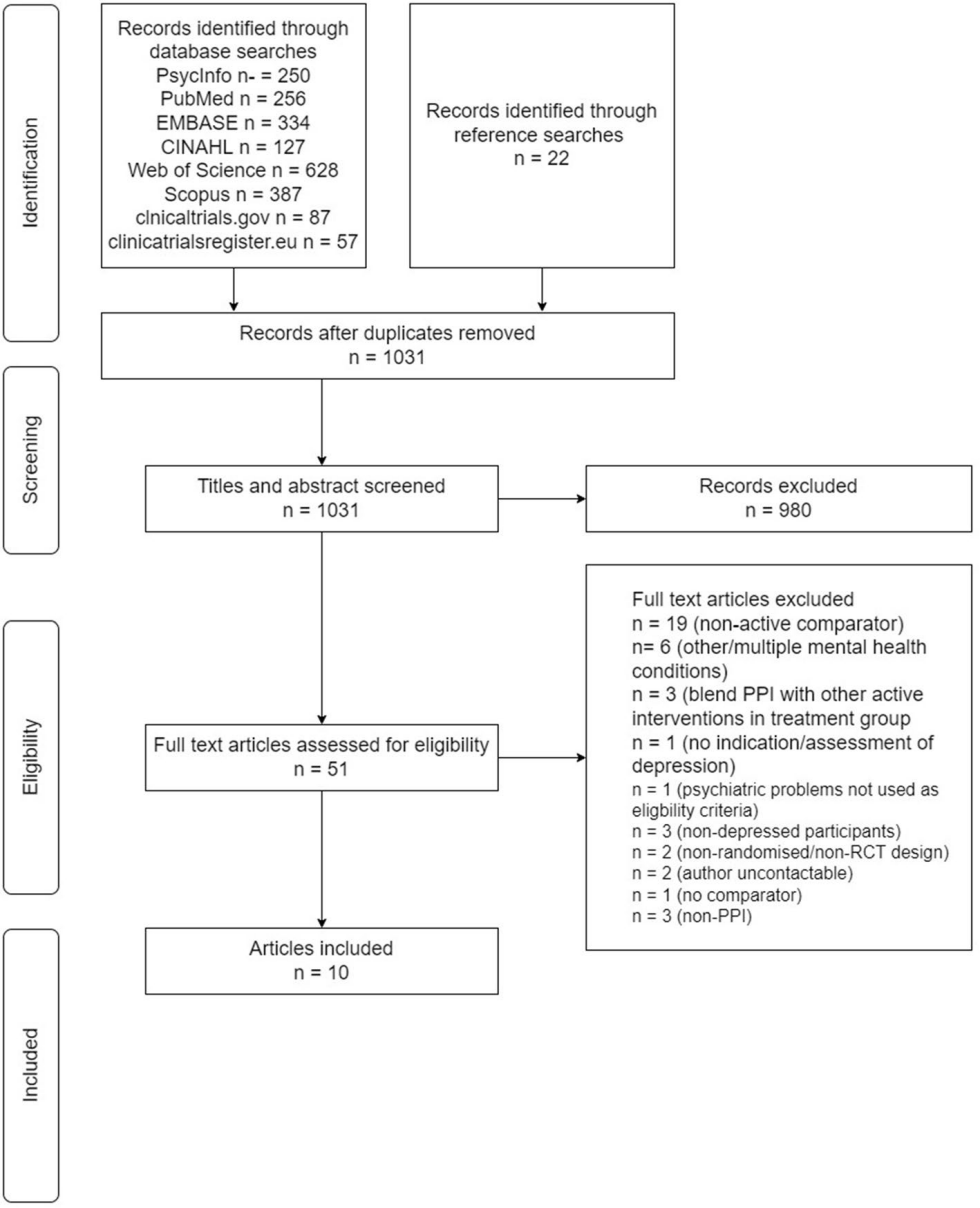
Flow diagram of study selection process

The search retrieved a total of 2148 results, of which 1982 were from databases, 144 from trial registers and 22 from searching the references of existing reviews. A total of 1031 references remained after the removal of duplicates. These were screened by title and abstract, which in turn led to 51 studies being selected for full text screening. The main reason for exclusion at the title and abstract screening stage was the use of no-treatment comparators. Full-text screening of the 51 studies resulted in ten being included in the review. The selection process is depicted in the flow diagram in Fig. 1.

One of the included studies, O’ Leary and Dockray (2015), did not specify that their participants were diagnosed or assessed as having depression. However, it can be inferred from the participants’ baseline depression scores that they were experiencing depression. The Edinburgh Postnatal Depression Scale (EDS) was used to assess depression in their participants. For this tool, a score of 10 and above indicates mild or major depression (Cox et al., 1987). In O’Leary and Dockray’s (2015) study, all three groups registered baseline scores of more than 20. The baseline means and standard deviations were 20.08 and 5.21 for the Gratitude group, 20.44 and 3.94 for the Mindfulness group, and 20.17 and 5.85 for the Control group. It is therefore apparent that the participants were experiencing depression at baseline, making the study eligible for review.

Another study, Uliaszek et al. (2016), included participants with multiple diagnoses. As the majority of the participants (71%) had a diagnosis of major depressive disorder or dysthymic disorder, changes in depression symptoms as measured by the Symptom Checklist-90 Revised (SCL-Dep) were likely due to this group of depressed participants. Therefore, the study was included, but only in the analysis for depression. It was not used for the analyses for well-being. This was because it is likely that participants with other diagnoses contributed to the well-being outcome as well, which would have contravened the review’s selection criteria.

### Study Characteristics

Table 1 presents the characteristics of the ten included studies. One was conducted in Iran (Asgharipoor et al., 2012), one was based in Canada but included globally diverse participants as its intervention was delivered online (Mongrain et al., 2016). The rest were conducted in Northern America and Europe. In total, the ten trials evaluated 1341 participants assessed to have mild to severe depression. There were 529 participants in the PPI arm and 812 in the comparator arm. The interventions were group-based in four of the trials. Out of the remaining six individualised interventions, four were self-administered and two were conducted by interventionists. One of these two was centre-based while the other was delivered over the phone. One study used only gratitude exercise as the PPI (O’Leary & Dockray, 2015), the rest included a variety of activities in their PPI. The most common comparator was CBT, used by five of the studies. This was not surprising as CBT is the treatment of choice for depression (National Institute for Health and Care Excellence, 2019). The comparators for the remaining five studies were mindfulness therapy, dialectic behavioural therapy, cognitive-focused exercises and unspecified active psychotherapies (“Treatment as usual”). Five studies (O’ Leary & Dockray, 2015; Mongrain et al., 2016; Celano et al., 2017; Furchtlehner et al., 2019; & Hanson, 2019) measured the outcomes at follow-up between two weeks and six months post-intervention, in addition to assessments at baseline and post-intervention. The other five studies measured the outcomes at baseline and post-intervention only (Asgharipoor et al., 2012; Chaves et al., 2017; Seligman et al., 2006; Uliaszek et al., 2016; Walker & Lampropoulos, 2014).

**Table 1:** Characteristics of included studies

Study	Country	Sample size	Participants	Depression status	Depression assessment	Intervention	Comparator	Outcome measures
Asgharipoor et al. (2012)	Iran	18	13 female 5 male Mean age 26.44 (SD 6.10) 9 Intervention 9 Comparator	Major depression	SCID	Group positive-oriented psychotherapy 2 h per week for 12 weeks	Group CBT 2 h per week for 12 weeks	Happiness (OHI, SWBS) Depression (BDI-II, SUDS)
Celano et al. (2017)	USA	65	45 female 20 male Mean age 44.0 (SD 16.6) 32 Intervention 33 Comparator	Primary diagnosis of major depressive disorder	MINI	Telephone positive psychology intervention 6 weeks One exercise per week	Cognitive-focused intervention 6 weeks One exercise per week	Hopelessness (BHS) Suicide Ideation (CHRTS) Depression (QIDS) Optimism (LOT-R) Gratitude (GQ) Positive Affect (PANAS)
Chaves et al. (2017)	Spain	96 Women	47 Intervention Mean age = 52..57 (SD = 9.38) 49 Comparator Mean age = 50.73 (SD = 11.34)	Major depression or dysthymia	SCID	Group PPI 2 h per week for 10 weeks	Group CBT 2 h per week for 10 weeks	Depression (BDI-II) Cognitive functioning (ATQ-30, RRS, WBSI) Emotional functioning (PANAS, BAI, RPA, DERS) Behavioural functioning (BIS/BAS) Well-Being (PHI, PWBS, SWLS, LOT-R, EOS)
Furchtlehner et al.( 2019)	Austria	92	59 female 33 male Mean age = 40.66 (SD = 12.4) 46 Intervention 46 Comparator	Mild to moderate depression Major depression in partial remission Dysthymia	SCID	Group manualised PPT (6 – 7 per group) 2 h per week for 14 weeks	Group manualized CBT 2 h per week for 14 weeks	Depression (BDI-II, DHS, MADRS) Overall psychological distress level (BSI)
Hanson (2019)	UK	115	100 female 15 male Mean age = 39.48 (SD = 11.32) 56 Intervention 59 Comparator	Symptoms of depression	BDI-II	Positive psychology self-help book Read 1 chapter per week for 8 weeks	CBT self-help book Read 1 chapter per week for 8 weeks	Depression (BDI-II) Well-Being (SHS, SWLS, PANAS, PWBS)
Mongrain et al. (2016)	Canada	741 globally diverse sample	478 female 263 male Mean age = 32.64 (SD 11.39) 265 Intervention 235 Comparator 241 Control	Mild to moderate depression	CES-D	Online positive activity Three weeks (practice 10 emotions, one emotion to be practiced every other day)	10-min online mindfulness meditation Three weeks (instructions delivered every other day)	Depression (CES-D) Subjective well-being (SWLS, OTH)
O’Leary and Dockray (2015)	Ireland	61 women	Mean age = 28.35 (SD = 6.65) 29 Intervention 22 Comparator 10 Control	Mild to major depression	EDS	Online gratitude exercises Four times per week for three weeks	Online Mindfulness exercise Four times per week for three weeks	Stress (PSS) Depression (EDS) Happiness (SHS)
Seligman et al. (2006)	US	32	22 female 10 male Age = 18 – 55 11 Intervention 9 Comparator 1 12 Comparator 2	Major Depression	DSM-IV	PPT 14 individual sessions over 12 weeks	Comparator 1 = TAU Comparator 2 = TAU + medications	Depression (ZSRS, HRSD) Global Improvement (OQ, GAF) Well-Being (PPTI, SWLS, self-created PPT outcome measure) Remission (ZSRS, HRSD, OQ, GAF)
Uliaszek et al. (2016)	Canada	35 women	Mean age before drop out (n = 54) = 22.17 (SD = 5.05) 13 Intervention 22 Comparator	A range of symptoms of psychopathology including major depression	SCID-I and SCID-II	12-week group PPT	12-week group DBT	Psychopathology symptoms (SCL-90R, LPI) Adaptive and maladaptive skills (DERS, DTS, KIMS, WOCCL) Well-Being (PPTI, SWLS)
Walker and Lampropoulos (2014)	US	86	59 female 27 male Mean age = 20.67 (SD = 2.49) 21 Intervention 21 Comparator 1 22 Comparator 2 22 Control	At least mild depression	CES-D	Positive psychology homework assignments 2 weeks Min 4 h per assignment/activity	Comparator 1 = CBT without interpersonal element Comparator 2 = CBT with interpersonal element 2 weeks Min 4 h per assignment/activity	Mental health symptoms (CES-D, OQ) Positive state (BADS, MHC-SF, PANAS)

ATQ-30 = Automatic Thoughts Questionnaire, BADS = Behavioral Activation for Depression Scale, BAI = Beck’s Anxiety Inventory, BDI-II = Beck’s Depression Inventory, second edition, BHS = Beck Hopelessness Scale, BIS/BAS = Behavioural Inhibition Scale and Behavioural Approach Systems Scale, BSI = Brief Symptom Inventory, CBT = Cognitive-Behavioural Therapy, CES-D = Centre for Epidemiological Studies Depression Scale, CF = Cognitive-Focused, CHRTS = Concise Health Risk Tracking Scale, DBT = Dialectic Behavior Therapy, DERS = Difficulties in Emotion Regulation Scale, DHS = Depression Happiness Scale, DSM = Diagnostic and Statistical Manual, DTS = Distress Tolerance Scale, EDS = Edinburgh Depression Scale, EOS = Enjoyment Orientation Scale, GAF = Global Assessment of Functioning, GQ = Gratitude Questionnaire, HRSD = Hamilton Rating Scale for Depression, KIMS = Kentucky Inventory of Mindfulness Skills, LOT-R = Life Orientation Test-Revised, LPI = Life Problems Inventory, MADRS = Montgomery Asberg Depression Rating Scale, MHC-SF = Mental Health Continuum – Short Form, MINI = Mini International Neuropsychiatric Interview, OHI = Oxford Happiness Inventory, OQ = Outcome Questionnaire, OTH = Orientations to Happiness Scale, PANAS = Positive and Negative Affect Schedule, PHI = Pemberton Happiness Index, PPI = Positive Psychology Intervention, PPT = Positive Psychotherapy, PPTI = Positive Psychotherapy Inventory, PSS = Perceived Stress Scale, PWBS = Ryff’s Psychological Well-Being Scales, QIDS = Quick Inventory of Depression Symptomatology, RPA = Response to Positive Affect Questionnaire, RRS = Ruminative Response Scale, SCID = Structured Clinical Interview for DSMIV, SCL-90R = Symptom Checklist-90 Revised, SHS = Subjective Happiness Scale, SUDS = Subjective Units of Depression Scale, SWLS = Satisfaction with Life Scale, TAU = Treatment as usual, WBSI = White Bear Suppression Inventory, WOCCL = DBT-Ways of Coping Checklist, ZSRS = Zung Self-Rating Scale

### Risk of Bias

The risk of bias assessment is summarised in Table 2. All studies presented unclear or high risk of bias for allocation concealment, performance bias, and reporting bias. Most did not report whether steps were taken to conceal group allocation from the participants (selection bias), or to blind participants to treatment (performance bias). Published protocols could not be found for the studies except for Furchtlehner et al. (2019), therefore reporting bias could not be ascertained. As for detection bias (assessment bias), only one study (Celano et al., 2017) reported using blinded assessors. The other nine studies clearly or very likely had participants self-completing most of the outcome assessment questionnaires. These studies were rated as presenting low risk of detection bias (Cook, 2010). It should be noted that in Furchtlehner et al.’s (2019) study, one of the investigators was involved in the intervention at one of their trial sites, potentially contributing to performance bias. The same investigator was also involved in data collection at the same site. However, as the data were collected via self-report, the risk of detection bias was still rated as low.

**Table 2:** Risk of bias assessment

	Random sequence generation (selection bias)	Allocation concealment (selection bias)	Blinding of participants and personnel (performance bias)	Blinding of outcome assessment (detection bias)	Incomplete outcome data (attrition bias)	Selective reporting (reporting bias)	Other bias
Asgharipoor et al. (2012)	Low	Unclear	Unclear	Low	Low	Unclear	Low
Celano et al. (2017)	Low	Low	Unclear	Low	Low	Unclear	Low
Chaves et al. (2017)	Low	Low	Unclear	Low	Low	Unclear	Low
Furchtlehner et al. (2019)	Low	Unclear	Unclear	Low	Low	High	Low
Hanson (2019)	Low	Unclear	Unclear	Low	Low	Unclear	High
Mongrain et al. (2016)	Low	Unclear	Unclear	Low	Unclear	Unclear	Unclear
O’Leary and Dockray (2015)	Low	Unclear	Unclear	Low	High	Unclear	Unclear
Seligman et al. (2006)	Low	Unclear	Unclear	Low	Unclear	Unclear	Low
Uliaszek et al. (2016)	Low	Unclear	Unclear	Low	Low	Unclear	Low
Walker and Lampropoulos (2014)	Low	Unclear	Unclear	Low	Low	Unclear	Low

### Selection of Outcome Measures

As shown in Table 1, the questionnaires used to measure well-being and depression differed from study to study, and all studies used multiple questionnaires to measure the outcomes. Among the different tools used to measure well-being, scales that measured happiness were consistently used in all the included studies. Therefore, based on the selection principle of conceptual convergence, happiness was chosen as the well-being variable to be meta-analysed. The other measures of well-being were too varied for the small number of studies.

As for depression, Walker and Lampropoulos (2014), Furchtlehner et al. (2019) and Seligman et al. (2006) used multiple measures, while the remaining studies used one measure. Beck’s Depression Inventory (BDI-II) was most commonly used (Asgharipoor et al., 2012; Chaves et al., 2017; Furchtlehner et al., 2019; Hanson, 2019), followed by the Centre for Epidemiological Study Depression Scale (CES-D) (Mongrain et al., 2016; Walker & Lampropoulos, 2014). Both BDI-II and CES-D were thus selected for the meta-analysis together with four other measures that were used individually by the remaining four studies (Seligman et al., 2006; O’ Leary & Dockray, 2015; Uliaszek et al., 2016; Celano et al., 2017).

### Meta-Analysis

#### Post-Intervention Effects

The meta-analyses results are summarised in Table 3. The effect for happiness favoured PPI but was not significant, Hedge’s g = 0.20 (95% CI = −0.12, 0.53), overall effect Z = 1.22, *p* = 0.22. There was substantial heterogeneity, Q = 30.40, *p* = 0.002; I^2^ = 74%. Similarly, for depression, the effect favoured PPI but was not statistically significant, Hedge’s g = 0.15 (95% CI = −0.19, 0.49), overall effect Z = 0.86, *p* = 0.39. Heterogeneity among the studies was also substantial, Q = 42.53, *p* = 0.00001, I^2^ = 79%. The results indicated no real difference in effectiveness between PPI and the comparators in either treating depression or increasing happiness.

**Table 3:** Summary meta-analysis results for depression and happiness

Outcome	Number of studies	Intervention sample size	Comparator sample size	Hedge’s g (95% CI)	Heterogeneity		Test for overall effect
					Q	I^2^	
Depression	10	494	462	0.15 (−0.19, 0.49)	42.53 df = 9 (*p* = 0.00001)	79%	Z = 0.86 (*p* = 0.39)
Happiness	9	467	435	0.20 (−0.12, 0.53)	30.40 df = 8 (*p* = 0.002)	74%	Z = 1.22 (*p* = 0.22)

#### Sensitivity Analysis

There was initial evidence of small studies effect in the meta-analyses. For both outcomes, the two smallest studies (Asgharipoor et al., 2012; Seligman et al., 2006) produced larger effect estimates than all the other studies except Furchtlehner et al. (2019). The meta-analyses were repeated with these two studies removed. Their removal did not significantly alter the results for either outcome. Higgins et al. (2019) recommended comparing fixed-effects and random-effects analyses when small studies effect is suspected and heterogeneity is present. Similar effect estimates between the two analyses implies that the small studies have little effect on the results. In this case, when the fixed-effects model was applied, the effect estimates for both outcomes became significant. However, the significant results were due to the disproportionate influence of the largest study (Mongrain et al., 2016). Therefore, although the fixed effects and random effects analyses produced different results, the results remain inconclusive.

As mentioned in the study selection section, two studies (O’ Leary & Dockray, 2015; Uliaszek et al., 2016) were included in the review although they did not fully meet the eligibility criteria. Thus, the meta-analyses were repeated with these two studies removed in turn. The results were not significantly altered, indicating that the addition of these two studies did not skew the results.

### Publication Bias

**Fig. 2:**
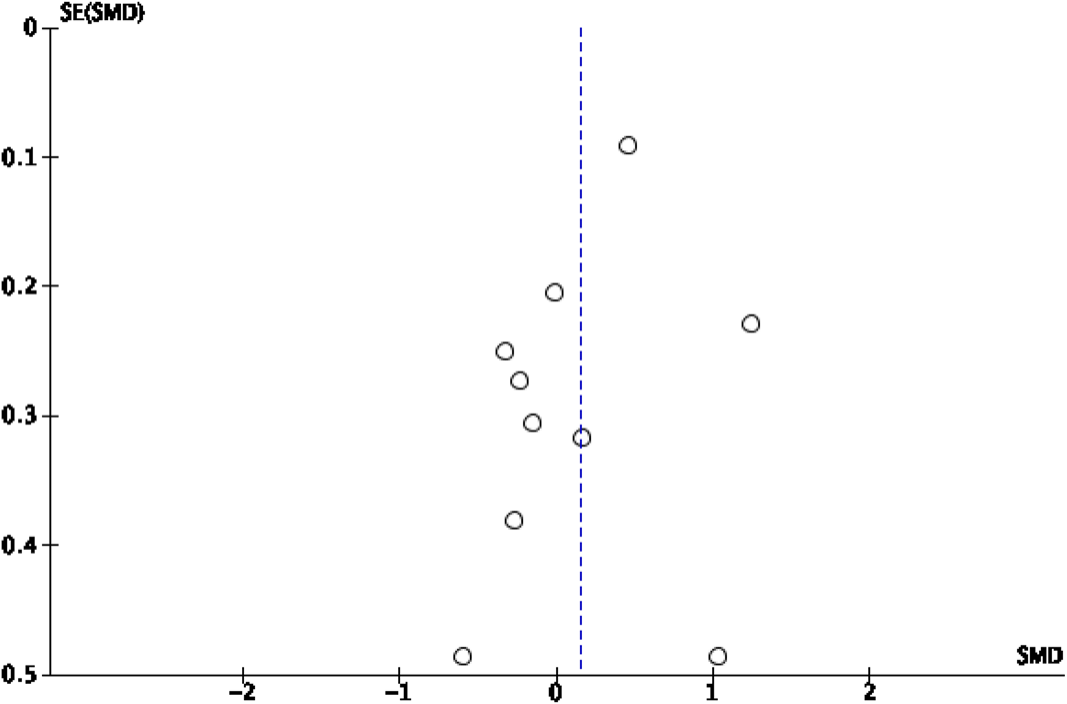
Funnel plot for depression

Figures 2 and 3 in the supplementary materials display the funnel plots of the two outcomes. Both funnel plots are asymmetrical—indicative of publication bias. The bias appears to be more pronounced for happiness than for depression, as the funnel plot for happiness deviates more from symmetry than depression. Specifically, the two smallest studies (Asgharipoor et al., 2012; Seligman et al., 2006) show moderately high precision of effect estimates for happiness, compared to depression for which these two studies reside on the base of the funnel plot, indicating low precision of effect estimates. Egger’s test was conducted to test the significance of the asymmetry. Both regression lines did not pass through the point of origin, indicating asymmetry. The intercept for happiness is below zero, revealing possible small studies effect (Egger et al., 1997). However, both the regression intercepts for happiness (intercept = −0.511, 93% CI = −1.812, 0.789) and depression (intercept = 0.363, 95% CI = −0.912, 1.638) were not significant. The results of the funnel plots and Egger’s tests have to be interpreted with caution. Sterne et al. (2011) recommended that funnel plots should only be done when there are at least ten studies, and this minimum number increases with higher heterogeneity. Therefore, the small number of studies and high heterogeneity may have likely limited the validity of the funnel plot. In a similar vein, Sterne et al. (2011) also advised against doing the Egger’s test if there are less than ten studies. Moreover, when there is substantial between-study heterogeneity, statistical tests for asymmetry tend towards being underpowered. For these reasons, publication bias could not be fully ascertained from the funnel plots and Egger’s tests. The asymmetries might more likely be due to heterogeneity, reporting bias and chance.

**Fig. 3:**
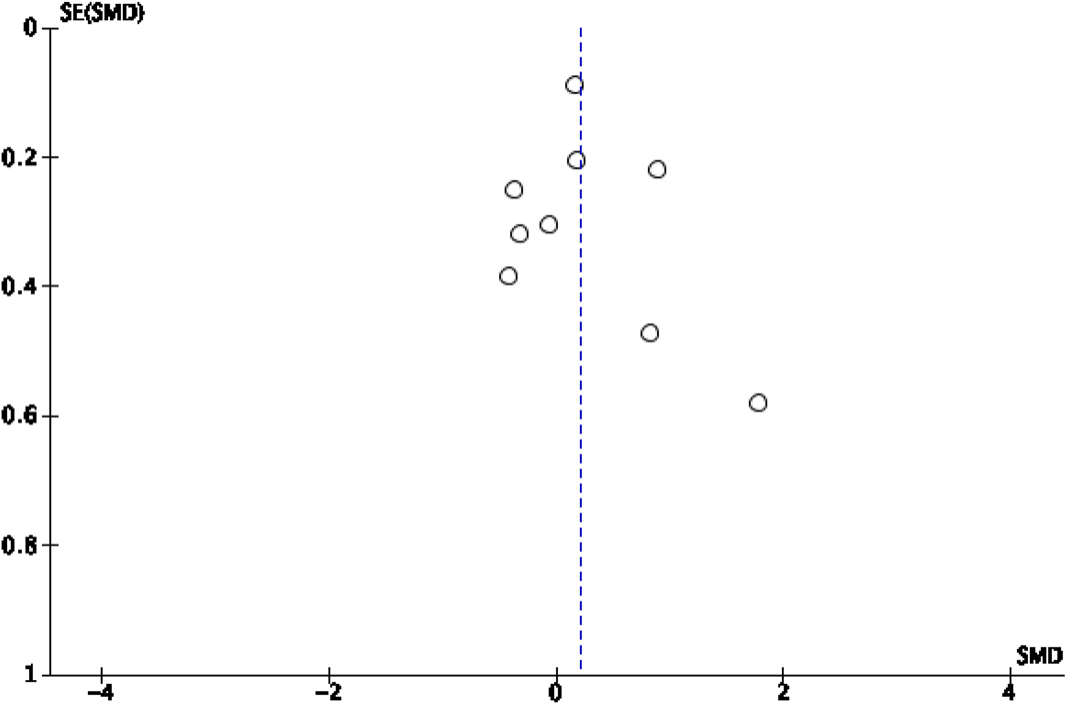
Funnel plot for happiness

## Discussion

This study systematically reviewed PPI’s effectiveness for increasing the well-being of depressed adults compared to other active psychological interventions. Its outcomes were improvement in well-being (happiness) and reduction in depression. It was hypothesised that firstly, PPI would fare better than other active psychological interventions for enhancing well-being, and secondly, PPI and other active psychological interventions would produce similar effects for reducing depression. The results supported the second but not the first hypothesis. The effect sizes of 0.20 for happiness (well-being) and 0.15 for depression were small and non-significant, suggesting no difference in effectiveness between PPI and other active interventions for reducing depression and enhancing well-being.

The effect size for happiness is the same as Bolier et al.’s (2013) 0.20 for psychological well-being, but smaller than other reviews (Carr et al., 2020; Hendriks et al., 2020; Chakhssi et al., 2018 and Sin & Lyubomirsky, 2009) where g = 0.28 to 0.39. The effect size of 0.15 for depression is smaller than that of the above reviews in which effect sizes ranged from 0.27 to 0.39. Moreover, the effects in those reviews were statistically significant, whereas this was not so for both effect sizes in the present review. This is not surprising given that the other reviews had many more included studies and a majority of non-treatment comparators.

With regards to comparing PPI with other active interventions, Carr et al.’s (2020) comparisons were statistically significant, g = 0.31 for well-being and g = −0.30 for depression; in contrast, effect sizes were not significant in the present review. Carr et al. (2020) had 226 studies that were highly mixed in study characteristics and study quality, which might have inflated the effect sizes. Comparatively, our review is closer to Geerling et al.’s (2020) in selection criteria and scope, and both reviews yielded non-significant results.

Contrary to the claim that PPI enhances well-being while other active interventions merely target depressive symptoms, our review did not find PPI to fare significantly better than active comparators in increasing happiness, despite the results favouring PPI. There could be a few possible reasons for this. It could be due to the small number of included studies and the modest sample sizes of most of those studies, making the review insufficiently powered to detect any significant effect. More and bigger trials are therefore needed. It could also be that common factors such as therapeutic alliance and patient expectancy (placebo effect) contributed more to the positive outcomes than the intervention itself (Ahn & Wampold, 2001).

At the same time, it could be that other active interventions are also capable of enhancing well-being. Two studies that examined CBT’s mechanism of change in treating panic disorder showed that increase in self-efficacy was a crucial step towards treatment efficacy (Fentz et al., 2013; Gallagher et al., 2013). This demonstrates the ability of CBT to not only alter faulty cognition but also cultivate positive cognition.

Perhaps the uniqueness of positive psychology in increasing positivity compared to other standard treatments holds true only under specific conditions. For instance, at present many psychological interventions focus on either remedying the past (e.g., psychoanalysis) or bettering the present (e.g., behavioural modification, mindfulness). Positive psychology contrasts with these interventions by accentuating the importance of being positive about not just the past and the present but also the future. Cultivating future-oriented positive cognition and emotion such as optimism and hope may be positive psychology’s unique contribution to mental health.

### Heterogeneity

There was substantial heterogeneity in both comparisons. This might occur from two main sources. The first was the wide variation in intervention implementation. There were assortments of group- and individual-based, in-person and online, as well as guided and self-help formats. For intervention content, there were single activity and multiple activities, as well as manualised and non-manualised activities. Furthermore, intervention duration and frequency differed from one trial to another. There were also within-trial individual differences in implementation. For example, Seligman et al., 2006 tailored their PPI activities according to an individual participant’s clinical needs, circumstances and feasibility of completing the activities. In Walker and Lampropoulos’ (2014) study, participants were allowed to decide how many activities to complete, with a minimum requirement of completing four activities. Participants were also asked to participate in self-chosen social and volunteering activities as part of the intervention, which would inevitably result in differences in how and where they went about completing the activities. Such differences distributed among a small number of studies would unavoidably give rise to sizeable heterogeneity. This heterogeneity can be gradually lessened when more studies adhere to uniform intervention format and content. However, doing so would compromise external validity, as calibrating an intervention according to patients’ characteristics and context is essential for psychological treatments.

The second source of heterogeneity could be the different ways in which well-being was operationalised and measured. To overcome this limitation, our review adopted the method of selecting measures that converged conceptually. It resulted in happiness being selected as the well-being measure for the meta-analysis. Other measures of well-being in the included studies were too varied. Going forward, researchers can consider adopting more common measures of well-being.

### Risk of Bias

The result of the risk of bias assessment did not differ much from other reviews of PPI (Bolier et al., 2013; Chakhssi et al., 2018; Hendriks et al., 2018). It revealed an overall moderate to high risk of bias. Table 2 shows that allocation concealment, blinding participants and personnel, blinding of outcome assessment, and reporting could be further tightened. Nevertheless, blinding interventionists and participants may not be possible in psychological interventions. In these situations, it may be reasonable for reviewers to consider removing these two criteria or scoring them as “Not Applicable”. The same can be said of blinding of outcome assessment when outcome assessments are self-administered (e.g., see Bolier et al., 2013; and Sutipan et al., 2017). Alternatively, reviewers can consider rating studies that use self-report as low on assessment bias, according to Cook’s (2010) argument that self-report minimises the risk of assessment bias.

### Implications for Mental Health Practice

Previous reviews (Bolier et al., 2013; Sin & Lyubomirsky, 2009) have suggested that people experiencing mild to moderate depression, as well as those whose depression is in remission, can benefit from PPI. Specifically, these reviews showed greater benefits when PPI is delivered at the individual level instead of in groups, when it is clinician-guided instead of self-administered, when it comprises multiple activities instead of a single activity, and when it is done over a longer rather than shorter period.

Until the present review, synthesis of evidence to examine whether PPI can be a viable substitute for traditionally preferred psychological treatments of depression was scarce. Our review adds to this body of knowledge by providing further evidence that PPI can be a low-intensity, low-cost replacement for traditionally preferred depression treatments. The results favoured PPI over the active comparators although they failed to attain statistical significance. However, the accumulative evidence attests to PPI’s prospects in benefitting people with mental health conditions who are unable to access standard treatments. The COVID-19 pandemic is an example of such a scenario.

COVID-19 has affected the world on an unprecedented scale in modern history. It is not just a health pandemic; it has also created a mental health pandemic (Waters et al., 2021). While more research is required to understand the long-term mental health impact of COVID-19, and the corresponding responses that are needed (Holmes et al., 2020), the current situation calls for research-supported interventions that can be implemented efficiently under pandemic conditions. This means interventions that can be self-directed or remotely guided, delivered on a large scale in a community, and are simple and easily accessible. Most importantly, besides maintaining and improving mental wellness, these interventions must provide people caught in a bleak situation with a sense of hope (Waters et al., 2021). PPI has much to offer in this respect, and is adaptable, simple to use and appropriate for self-help.

However, reviews by Bolier et al. (2013) and Sin and Lyubomirsky’s (2009) showed that PPI is less effective when self-administered compared to clinician-guided, individual sessions. More precise studies are needed to compare the usefulness of self-administered and clinician-guided PPI because there may be other factors that better predict PPI effectiveness, such as patient motivation and commitment. Self-administered PPI may suit patients who value flexibility while clinician-guided therapy may work better for those who require structure and accountability.

Nevertheless, on this note, Layous et al. (2011) argued that self-administration is more feasible in situations where there is a need for widespread implementation of an intervention, such as during a pandemic lockdown. Therefore, self-administered PPI is still worthwhile in situations where access to guided interventions is limited. Moreover, Bolier et al. (2013) added that an intervention with a small effect can still create a sizable impact when there is a wide reach. More importantly, Bolier et al. (2013) also noted the need to study how to increase the effectiveness of self-help PPI, as doing so is well-aligned with positive psychology’s aim for PPI to be self-directed for most people. In addition, hybrid delivery modes can also be explored, such as a self-administered PPI with scheduled clinician check-ins.

### Limitations

The number of studies included in the review was small, and the sample sizes in many of these studies were modest. As a result, the analyses were underpowered to produce significant effects, thus precluding any firm conclusions. More and bigger trials are needed to add to its findings. Secondly, the substantial heterogeneity and presence of biases could have resulted in overestimation of the effects. Future trials may want to explore ways to decrease heterogeneity and bias. Thirdly, despite conducting a more extensive search compared to previous reviews, publication bias could not be ruled out because the search strategy did not include studies published in non-English languages and grey literature. Fourthly, not all studies conducted follow-up outcome assessments, hence the intermediate to long-term sustainability of the outcomes could not be fully evaluated. Furthermore, there was insufficient data to examine moderator effects, which could otherwise have shed more light on whether PPI worked better for certain groups of patients or under certain conditions. Nevertheless, moderator analyses conducted by previous reviews have provided considerable insights into the issue (e.g., Boiler et al., 2013; Sin & Lyubomirsky, 2009). Finally, only one author conducted the screening of studies, although a second reviewer checked the included and excluded studies. This could pose a potential source of bias as there was a lack of a second reviewer to conduct the screening of studies independently.

### Future Research

In response to the limitations, improvements are possible in future research. Firstly, heterogeneity could be reduced by implementing manualised PPI such as the Positive Psychotherapy programme (Seligman et al., 2006; Rashid, 2015). Another way to reduce heterogeneity is to use conceptually similar measures to assess the outcomes. Secondly, in terms of bias, although blinding interventionists and participants cannot be realistically done in most situations, other criteria can still be ensured by implementing trials more scrupulously and reporting them more thoroughly. Specifically, areas that can be strengthened include detailed reporting of random sequence generation, implementing allocation concealment and reporting the procedure, and publishing trial protocols. For assessment bias, when self-report is used or assessors cannot be blinded, it is important to ensure that standardised and psychometrically validated measures are used, and that assessors are properly trained to conduct the assessment (de Oliveira Souza et al., 2016; Sedgwick, 2015). There is also a need for more trials that conduct follow-up assessments to examine the intermediate to long-term sustainability of the effects. Finally, future trials can look into PPI’s mechanisms of change, besides merely studying whether it works. Lyubomirsky and Layous (2013) proposed a person-activity fit model that spells out activity- and person-related factors that moderate the effectiveness of positive activities on well-being. The authors asserted that the best result is achieved when there is an optimal fit between person and activity. Future trials could set out to test and refine Lyubomirsky and Layous’ (2013) model.

## Conclusion

The results of this systematic review did not support the claim that PPI enhances well-being while other standard interventions merely treat depressive symptoms. It found no difference in effectiveness between PPI and other active interventions for improving the well-being of adults experiencing depression. However, due to various limitations, its findings are inconclusive. More studies are needed to accrue the evidence in this area. Nevertheless, the findings suggest PPI’s potential as a viable alternative that has the same outcomes as other psychological interventions.

## Supplementary Information

Below is the link to the electronic supplementary material.

Supplementary file 1 (DOC 64 KB)

Supplementary file 2 (DOCX 14 KB)
