# Supplementary material for: The Effectiveness of Positive Psychology Interventions for Promoting Well-being of Adults Experiencing Depression Compared to Other Active Psychological Treatments: A Systematic Review and Meta-analysis
Source: J Happiness Stud. 2022 Nov 5;24(1):249–73. doi: 10.1007/s10902-022-00598-z (PMC9638203; doi:10.1007/s10902-022-00598-z)
Supplement: Supplementary file 2 — (DOCX 14 KB) [file 10902_2022_598_MOESM2_ESM.docx]

## CINAHL

#1 AB“positive psychology” OR AB“positive psychotherapy”

#2 AB gratitude OR AB blessings OR AB optimism OR AB compassion* OR AB forgiv* OR AB kind* OR AB meaning* OR AB “character strength” OR AB “signature strength”

#3 combine #1 or #2

#4 AB depress* OR AB “mood disorder” OR AB “affective disorder”

#5 AB (well-being or wellbeing or well being) OR AB happiness OR AB happy

#6 AB random* OR AB trial* OR AB RCT*

#7 combine #3 and #4 and #5 and #6

## EMBASE

#1 (“positive psychology” or “positive psychotherapy”).ab.

#2 (gratitude or blessings or optimism or compassion* or kind* or meaning* or “character strength” or “signature strength” or forgiv*).ab.

#3 combine #1 or #2

#4 (depress* or “mood disorder” or “affective disorder”).ab.

#5 (well-being or wellbeing or “well being” or happy or happiness).ab.

#6 (random* or trial* or RCT*).ab.

#7 combine #3 and #4 and #5 and #6

## PsycInfo

#1 (“positive psychology” or “positive psychotherapy”).ab.

#2 (gratitude or blessings or optimism or compassion* or kind* or meaning* or “character strength” or “signature strength” or forgiv*).ab.

#3 combine #1 or #2

#4 (depress* or “mood disorder” or “affective disorder”).ab.

#5 (well-being or wellbeing or “well being” or happy or happiness).ab.

#6 (random* or trial* or RCT*).ab.

#7 combine #3 and #4 and #5 and #6

## PubMed

#1 (“positive psychology”[Title/Abstract]) OR “positive psychotherapy”[Title/Abstract]

#2 ((((((((grattitude[Title/Abstract]) OR blessings[Title/Abstract]) OR optimism[Title/Abstract]) OR compassion*[Title/Abstract]) OR kind*[Title/Abstract]) OR forgiv*[Title/Abstract]) OR meaning*[Title/Abstract]) OR “character strength”[Title/Abstract]) OR “signature strength” [Title/Abstract]

#3 ((depress*[Title/Abstract]) OR “mood disorder”[Title/Abstract]) OR “affective disorder” [Title/Abstract]

#4 (((well-being[Title/Abstract]) OR wellbeing[Title/Abstract] OR “well being” [Title/Abstract] OR happy[Title/Abstract]) OR happiness[Title/Abstract]

#5 ((random*[Title/Abstract]) OR trial*[Title/Abstract]) OR RCT*[Title/Abstract]

#6 (((“positive psychology”[Title/Abstract]) OR “positive psychotherapy”[Title/Abstract])) OR (((((((((grattitude[Title/Abstract]) OR blessings[Title/Abstract]) OR optimism[Title/Abstract]) OR compassion*[Title/Abstract]) OR kind*[Title/Abstract]) OR forgiv*[Title/Abstract]) OR meaning*[Title/Abstract]) OR “character strength”[Title/Abstract]) OR “signature strength” [Title/Abstract])

#7 (((((((“positive psychology”[Title/Abstract]) OR “positive psychotherapy”[Title/Abstract])) OR (((((((((grattitude[Title/Abstract]) OR blessings[Title/Abstract]) OR optimism[Title/Abstract]) OR compassion*[Title/Abstract]) OR kind*[Title/Abstract]) OR forgiv*[Title/Abstract]) OR meaning*[Title/Abstract]) OR “character strength”[Title/Abstract]) OR “signature strength” [Title/Abstract]))) AND (((random*[Title/Abstract]) OR trial*[Title/Abstract]) OR RCT*[Title/Abstract])) AND (((((well-being[Title/Abstract]) OR wellbeing[Title/Abstract] OR “well being” [Title/Abstract] OR happy[Title/Abstract]) OR happiness[Title/Abstract])) AND (((depress*[Title/Abstract]) OR “mood disorder”[Title/Abstract]) OR “affective disorder” [Title/Abstract])

## Scopus

#1 (ABS (“positive psychology”) OR ABS (“positive psychotherapy”))

#2 (ABS (gratitude) OR ABS (blessings) OR ABS (optimism) OR ABS (forgiv*) OR ABS (compassion*) OR ABS (meaning*) OR ABS (kind*) OR ABS (“character strength”) OR ABS (“signature strength”))

#3 ((ABS (“positive psychology”) OR ABS (“positive psychotherapy”))) OR ((ABS (gratitude) OR ABS (blessings) OR ABS (optimism) OR ABS (forgiv*) OR ABS (compassion*) OR ABS (meaning*) OR ABS (kind*) OR ABS (“character strength”) OR ABS (“signature strength”)))

#4 (ABS (depress*) OR ABS (“mood disorder”) OR ABS (“affective disorder”))

#5 (ABS (well-being) OR ABS (wellbeing) OR ABS (well being) OR ABS (happiness) OR ABS (happy))

#6 (ABS (random*) OR ABS (trial*) OR ABS (rct*))

#7 (((ABS (“positive psychology”) OR ABS (“positive psychotherapy”))) OR ((ABS (gratitude) OR ABS (blessings) OR ABS (optimism) OR ABS (forgiv*) OR ABS (compassion*) OR ABS (meaning*) OR ABS (kind*) OR ABS (“character strength”) OR ABS (“signature strength”)))) AND ((ABS (depress*) OR ABS (“mood disorder”) OR ABS (“affective disorder”))) AND ((ABS (well-being) OR ABS (wellbeing) OR ABS (well being) OR ABS (happiness) OR ABS (happy))) AND ((ABS (random*) OR ABS (trial*) OR ABS (rct*)))

## Web of Science

#1 **TOPIC:** (“positive psychology”) *OR* **TOPIC:** (“positive psychotherapy”)

#2 **TOPIC:** (gratitude) *OR* **TOPIC:** (blessings) *OR* **TOPIC:** (optimism) *OR* **TOPIC:** (compassion*) *OR* **TOPIC:** (forgiv*) *OR* **TOPIC:** (meaning*) *OR* **TOPIC:** (kind*) *OR* **TOPIC:** (“character strength”) *OR* **TOPIC:** (“signature strength”))

#3 combine #2 OR #1

#4 **TOPIC:** (depress*) *OR* **TOPIC:** (“mood disorder”) *OR* **TOPIC:** (“affective disorder”))

#5 **TOPIC:** (well-being) *OR* **TOPIC:** (wellbeing) *OR* **TOPIC:** (well being) *OR* **TOPIC:** (happiness) *OR* **TOPIC:** (happy)

#6 **TOPIC:** (random*) *OR* **TOPIC:** (trial*) *OR* **TOPIC:** (RCT*)

#7 combine #6 AND #5 AND #4 AND #3
